# From pupil to the brain: New insights for studying cortical plasticity through pupillometry

**DOI:** 10.3389/fncir.2023.1151847

**Published:** 2023-03-31

**Authors:** Aurelia Viglione, Raffaele Mazziotti, Tommaso Pizzorusso

**Affiliations:** ^1^BIO@SNS Lab, Scuola Normale Superiore, Pisa, Italy; ^2^Institute of Neuroscience, National Research Council, Pisa, Italy

**Keywords:** pupillometry, pupil size, locus coeruleus, norepinephrine, noradrenaline, neuromodulation, cortical plasticity

## Abstract

Pupil size variations have been associated with changes in brain activity patterns related with specific cognitive factors, such as arousal, attention, and mental effort. The locus coeruleus (LC), a key hub in the noradrenergic system of the brain, is considered to be a key regulator of cognitive control on pupil size, with changes in pupil diameter corresponding to the release of norepinephrine (NE). Advances in eye-tracking technology and open-source software have facilitated accurate pupil size measurement in various experimental settings, leading to increased interest in using pupillometry to track the nervous system activation state and as a potential biomarker for brain disorders. This review explores pupillometry as a non-invasive and fully translational tool for studying cortical plasticity starting from recent literature suggesting that pupillometry could be a promising technique for estimating the degree of residual plasticity in human subjects. Given that NE is known to be a critical mediator of cortical plasticity and arousal, the review includes data revealing the importance of the LC-NE system in modulating brain plasticity and pupil size. Finally, we will review data suggesting that pupillometry could provide a quantitative and complementary measure of cortical plasticity also in pre-clinical studies.

## Introduction

Neural plasticity refers to the ability of neural circuits to adapt and change in response to internal or external stimuli. This ability allows neurons to adjust their molecular, physiological, and morphological characteristics to respond quickly to salient environmental changes. For appropriate responses to be executed, there must be a rapid reorganization of the neural networks, resulting in increased or decreased activity across a significant portion of the brain. The neuromodulator norepinephrine (NE) plays an important role in optimizing these responses. The locus coeruleus (LC), a small bilateral nucleus located in the brainstem, is the primary source of NE in the brain, (Poe et al., 2020) with broad projections that pervade the cortex (Foote et al., 1983). Most neural innervation in the cerebral cortex involving NE is non-synaptic, with molecules diffusing to nearby receptors. The diffuse release of NE into the extracellular space is consistent with its role as a neuromodulator and its wide range of effects on various cellular targets within the cerebral cortex (Séguéla et al., 1990). Moreover, the NE-system functioning depends on the expression of different receptors in both neurons and glial cells throughout the central nervous system (CNS) (O’Donnell et al., 2012). This complexity enables the LC-NE system to induce significant changes in neuronal activity, network connectivity and to mediate a broad spectrum of brain functions, comprising wakefulness (Berridge and Waterhouse, 2003), arousal, and high-order processes (e.g., attention, sensory processing, and learning) (McBurney-Lin et al., 2019). Experiments in adult mice suggest that NE is necessary for inducing changes in the receptive fields of cortical sensory circuits, while brief NE increases alter neuronal tuning (Manunta and Edeline, 2004; Shepard et al., 2015). The LC-NE system has the potential to induce brain plasticity through multiple functional mechanisms. For instance, when an arousing or emotionally significant stimulus is presented, the LC discharges a burst of NE throughout the brain, enhancing the sensitivity of sensory responses to particular environmental features, and modifying the overall network reactivity (Marzo et al., 2009).

It is commonly accepted that alterations in pupil size can serve as a reliable indicator of activity in the LC, and fluctuations in pupil diameter are thought to occur simultaneously with the release of NE (Reimer et al., 2016). Pupillometry, the study of variation in pupil diameter, is emerging as a promising tool to directly assess the LC-NE system activity. Although changes in pupil size are mainly influenced by light, they may also serve as an indicator of cognitive processes and arousal states (Kahneman and Beatty, 1966; Schmidt and Fortin, 1982; Granholm and Steinhauer, 2004; Nassar et al., 2012). Higher cognitive and emotional processes can evoke tonic or phasic pupillary changes in humans and animal models (Lee and Margolis, 2016; Krebs et al., 2018). In humans, pupillary dilations can be induced by endogenous factors such as attention level, memory load, decision making and emotional processing (Kahneman and Beatty, 1966; Bradley et al., 2008; Wierda et al., 2012; Binda et al., 2013; de Gee et al., 2014; Lisi et al., 2015). In mice, fluctuations in pupil constriction and dilation have been shown to reliably reflect the sensory responsiveness of the cortex to different stimuli (Lee and Margolis, 2016). In both mice and humans, changes in pupil size are known to be linked to arousal and vigilance levels (Murphy et al., 2011; Yüzgeç et al., 2018; Martin et al., 2022).

Recent advancements in hardware technology and software development have made it possible to measure pupil size in a variety of experimental settings accurately (Mazziotti et al., 2021; Privitera et al., 2021), leading to an increased interest in using pupillometry as a tool for understanding the activity of the nervous system and, potentially, as a biomarker for brain disorders. However, the potential of pupillometry to provide insights into the role of the LC-NE system in neural processes and plasticity needs to be better understood. In this minireview, we will focus on the evidence supporting the importance of the LC-NE system in modulating brain plasticity. Given the close relationship between LC-NE tone and pupil size (Joshi et al., 2016), we will also explore pupillometry as a non-invasive method for studying cortical plasticity.

## An overview of the LC-NE system

**FIGURE 1:**
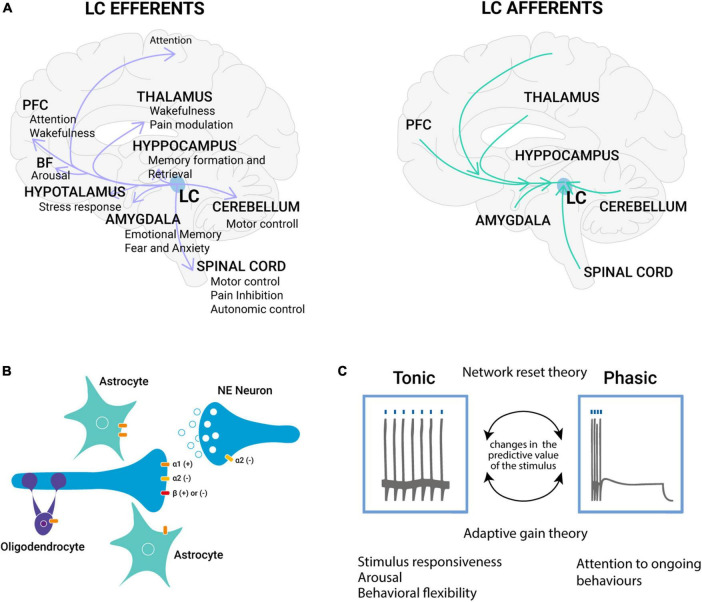
The noradrenergic system complexity: from molecules to function. **(A)** LC efferent and afferent projections. The LC is a small bilateral nucleus in the brainstem that widely innervates the brain with broad projections that pervade the cortex. All LC neurons contain NE, thus the LC serves as the primary source of NE in the forebrain. NE released from LC is a ubiquitous neuromodulator that has been linked to multiple functions, including arousal, action sensory gain, and learning (Szabadi, 2013). **(B)** Diffuse hormone-like action of NE release. NE α1- and β-receptors are thought to exist primarily post-synaptically (heteroreceptors), whereas α2-receptors are present both pre- (autoreceptors) and post-synaptically (Szabadi and Bradshaw, 1991). The autoreceptors of the α2 sub-class are inhibitory in action and are involved in the inhibition of neuronal firing (Bertolino et al., 1997; Huang et al., 2012) or NE release (Starke, 2001) based on their location. α1-receptors are mainly excitatory, while β-receptors are excitatory or inhibitory (Szabadi, 2013). In the CNS, adrenergic heteroreceptors have been identified on the terminals of serotonergic (Haj-Dahmane and Shen, 2014), dopaminergic (Mejias-Aponte, 2016), and glutamatergic (Mori-Okamoto et al., 1991) neurons, extending its action on different classes of neurons well known to be involved in brain plasticity. Moreover, central noradrenergic neurons also make contacts with the CNS’s non-neuronal elements, such as glial cells. **(C)** Tonic and phasic LC activity. Two theories are trying to explain the bimodal spiking activity of LC, the adaptive gain theory, proposed by Aston-Jones and Cohen (2005) posits that phasic LC activity prevails during exploitation, facilitating task-specific decision processes; tonic activity instead prevails during periods of exploration, making targeted circuits more responsive to any stimulus. However, this theory does not explain which phenomenon is responsible for the shift from the two phases. A second theory, named network reset theory, posits that the NE signal would have a general reset function. In particular, it has been demonstrated that LC responses undergo habituation in the absence of reinforcement (Vankov et al., 1995), while when the predictive value of the stimulus is reversed, habituated LC neurons respond to the change. PFC, prefrontal cortex; BF, basal forebrain; LC, locus coeruleus; NE, norepinephrine.

The LC is a small nucleus located in the dorsal tegmentum with a high level of complexity in terms of its molecular, cellular, and regional targets. As all neurons in the LC contain NE, the LC serves as the foremost source of NE in the forebrain, projecting widely to both cortical and subcortical regions (Szabadi, 2013; Benarroch, 2018; Figure 1A). The extensive distribution of noradrenergic fibers in the neocortex suggests that the projections originating from the LC broadly impact the neocortex (Morrison et al., 1979). This extensive LC modulation of the cortex underlies the role of the LC in controlling brain state, such as arousal (Carter et al., 2010; Sara and Bouret, 2012), locomotion (Polack et al., 2013), exploration (Gompf et al., 2010), and attention (Bouret and Sara, 2004). In addition to NE, noradrenergic neurons also release various co-transmitters, including glutamate (Yang et al., 2021), ATP (Poelchen et al., 2001), neuropeptide Y (Tsuda et al., 1989; Illes and Regenold, 1990), the neuropeptide galanin (Tillage et al., 2020, 2021), and dopamine (Devoto and Flore, 2006; Kempadoo et al., 2016). The co-transmitters could modulate the action of NE both pre-synaptically and post-synaptically, with effects on NE release and neurotransmission (Burnstock, 2009; Herring and Paterson, 2009). Although some noradrenergic boutons form direct synaptic contacts with neurons, many are primarily non-synaptic without any identifiable synaptic connection. This particular characteristic of central noradrenergic neurons implies that NE may also exert more widespread hormonal effects throughout the brain (Beaudet and Descarries, 1978; Descarries and Mechawar, 2000; Figure 1B). Studies have shown that NE is diffusely released in various structures of the CNS, including the amygdala (Zhang et al., 2013), the hypothalamus (Michaloudi et al., 1997), and the cerebral cortex (Agster et al., 2013). This supports the idea that the LC-NE system plays a role in the coordinated regulation of large brain regions in response to significant stimuli. Central noradrenergic neurons make contacts with neurons and with non-neuronal elements of the CNS, such as glial cells (Figure 1B). Astrocytes may act as intermediaries of NE impact on neuronal activity. When glial adrenergic receptors are activated, astrocytes experience a quick increase in calcium levels which enhances synaptic plasticity (Gordon et al., 2005) or increases cAMP levels which modulate the process of memory consolidation (Oe et al., 2020).

The effectiveness of the noradrenergic system is reliant on the distinct expression of various receptor types in both neurons and glial cells. There are three adrenoceptors families, β, α1, and α2, each composed of multiple subtypes (Perez, 2020). The adrenoreceptors are all known to be metabotropic receptors, and the affinity to NE is higher for the α-receptors than for the β-receptors (Wu et al., 2021; Figure 1B). The LC is characterized by a phasic and a tonic activity mode (Vazey et al., 2018; Figure 1C). The “adaptive gain theory,” proposed by Aston-Jones and Cohen (2005), tries to explain this bimodal activation of the LC-NE system (Aston-Jones and Cohen, 2005). According to this theory, phasic activity is driven by decision processes related to the task at hand and facilitates performance optimization (Clayton et al., 2004). In contrast, during withdrawal from the current task and the beginning of alternative behaviors, tonic activity tends to prevail (Aston-Jones and Cohen, 2005; Figure 1C). By employing “adaptive gain,” LC-NE activity enhances the balance between focused and flexible behaviors by alternating between phasic and tonic activity modes (Mathôt, 2018). Nonetheless, it remains unclear which internal or external triggers prompt the LC to shift between these two spiking pattern modes. The “network reset theory,” which constitutes a second hypothesis, proposes that NE signals facilitate the dynamic reorganization of targeted neural networks, enabling swift behavioral adaptation to changing environmental demands (Bouret and Sara, 2005). In particular, Vankov et al. (1995) demonstrated that LC responses habituate in the absence of reinforcement (Vankov et al., 1995). However, when the stimulus-reinforcement relationship changes or the predictive value of the stimulus is reversed, habituated LC neurons respond to the change (Bouret and Sara, 2004). These findings indicate that LC neurons exhibit a response to task-relevant stimuli in situations where their incidence cannot be entirely predicted (unexpected uncertainty), leading to a “reset in network activity” to facilitate updating prior probabilities (Yu and Dayan, 2005; Dayan and Yu, 2006). These two theories are not mutually exclusive and overlap with the role of NE in promoting cognitive shifts. Due to a strong correlation between LC activity and pupil size (Joshi et al., 2016), pupillometry has become a popular method for investigating the phasic and tonic modes of LC-NE activity. Phasic LC activity is linked to intermediate pupil size, while high tonic LC activity is associated with large pupils. Conversely, low LC activity and small pupils are indicative of sleepiness (Mathôt, 2018).

The strong relationship between LC activity and pupil size provides an attractive opportunity to use a straightforward and non-invasive physiological measure for assessing the activity of neuromodulators such as NE in specific regions of the brain.

## Neuromodulatory control of pupil size

**FIGURE 2:**
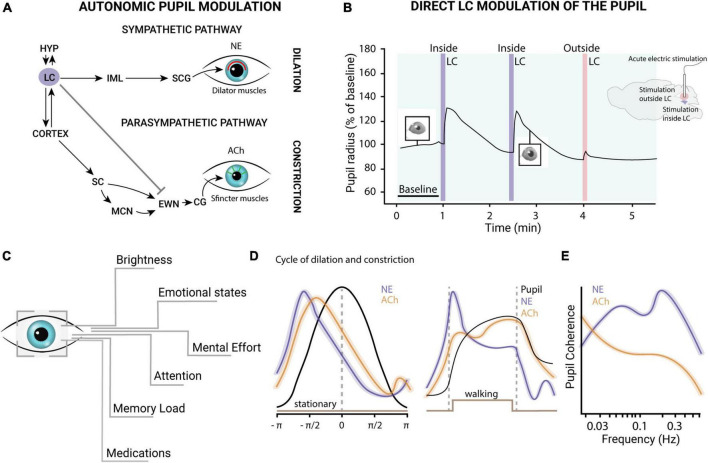
**(A)** The pupil constriction and dilation pathways. The iris of the eye contains two muscles that control its size: the sphincter muscle and the dilator muscle. The size of the iris is regulated by two interconnected neural pathways: the parasympathetic constriction pathway and the sympathetic dilation pathway. The parasympathetic constriction pathway originates in the EWN, which *via* cholinergic preganglionic motoneurons, sends its axons to synapse on the sphincter muscle. This pathway causes sphincter muscle contraction, leading to constriction of the pupil. The sympathetic dilation pathway originates in the SCG and sends axons to synapse on the dilator muscle. This pathway causes the dilator muscle relaxation, leading to pupil dilation. The LC plays a key role in regulating the sympathetic nervous system, including the sympathetic dilation pathway that controls pupil size. The LC is responsible for releasing the neurotransmitter NE, which acts on the dilator muscle and regulates pupil size. Activation of the LC leads to the dilation of the pupil, and inhibition of the LC leads to constriction of the pupil. Additionally, studies have shown that the LC-NE system regulates the pupillary light reflex, which is the automatic response of the pupil to changes in light intensity. **(B)** LC-pupil relationship. The acute electric stimulation of the LC is able to evoke pupillary dilations in mice. Redrawn from Privitera et al. (2021). **(C)** Exogenous factors influencing pupil size. **(D)** The left panel shows ACh (orange) and NE (violet) dynamics during the dilation (values < 0) and constriction (values > 0) phases in the absence of locomotion. The right panel illustrates NE and ACh activity during locomotion onset and offset. NE activity levels were higher and had a shorter latency than ACh activity preceding the peak of dilation. These findings suggest that both neuromodulatory systems contribute to regulating pupil size changes during quiet wakefulness, with NE playing a more prominent role in rapid and transient pupil responses. During locomotion, phasic noradrenergic axonal activity (violet) is closely linked to rapid pupil dilations, while sustained cholinergic axonal activity (orange) is associated with longer-lasting dilations (left). This figure is adapted from Reimer et al. (2016). **(E)** Coherence of NE and ACh in pupillary oscillations: NE levels display coherence with pupillary fluctuations across a wide frequency range. In contrast, ACh exhibits coherence primarily at lower frequencies, indicating distinct roles of the two neuromodulatory systems in the initiation and maintenance at different time scales. This figure is adapted from Reimer et al. (2016). HYP, hypothalamus; LC, locus coeruleus; SC, superior colliculus; MCN, mesencephalic cuneiform nucleus; EWN, Edinger-Westphal nucleus; CG, ciliary ganglion; ACh, acetylcholine; IML, intermediolateral cell column of the spinal cord; SCG, superior cervical ganglion; NE, norepinephrine.

Our pupils undergo a continuous fluctuation in size in response to variations in ambient light levels to regulate the amount of light that reaches the retina to optimize visual performance. However, even under isoluminant conditions, pupil size can be modulated by attention, working memory, perceptual and cognitive processes (Kahneman and Beatty, 1966; Schmidt and Fortin, 1982; Granholm and Steinhauer, 2004; Nassar et al., 2012). Two sets of muscles control pupil size: the iris sphincter muscle, which constricts the pupil, and the iris dilator muscle, which promotes dilation. These two muscles are controlled, respectively, by the parasympathetic constriction pathway and the sympathetic dilation pathway (Figure 2A). In the pupillary light reflex (PLR), the retina encodes and transmits changes in light levels to the brainstem pretectal olivary nucleus, which mainly controls pupil size *via* projections to the Edinger–Westphal nucleus (EWN). The EWN nucleus contains cholinergic preganglionic motoneurons that regulate the iris sphincter muscle. This preganglionic motoneurons project to the ciliary ganglion of the third cranial nerve, which controls the iris sphincter muscle through the ciliary nerve. The activity of projecting neurons in the EWN nucleus triggers the contraction of the iris sphincter muscle and constriction of the pupil. Conversely, inhibition of EWN neurons causes relaxation of the iris sphincter muscle, leading to dilation. The dilation pathway, instead, is a subcortical pathway that originates in the hypothalamus and the LC and connects to the iris dilator muscle (Figure 2A).

There is extensive evidence of functional relationships between LC activity and pupil dilation. In humans, fMRI studies combined with pupillometry measures have shown that LC activity increases together with pupil size during behavioral tasks and in resting (de Gee et al., 2017). Another fMRI study, conducted on humans performing an oddball task, pupil size changes have been described to covary with blood-oxygen-level-dependent (BOLD) signal localized to LC (Murphy et al., 2014). Moreover, the electrical stimulation of LC in anesthetized and awake animals evokes pupil dilation (Joshi et al., 2016; Reimer et al., 2016; Privitera et al., 2021; Figure 2B). These data support the idea of a direct coupling between the LC and pupil diameter. The LC could act directly on neurons in the EWN, with NE (Breen et al., 1983) binding the inhibitory α2-adrenergic receptors (Koss, 1986). However, the existence of a direct pathway is still controversial (Nieuwenhuis et al., 2011).

A recent study has investigated the accuracy by which pupil size can be used to index LC activity in mice (Megemont et al., 2022). The authors recorded spiking activity from LC neurons optogenetically tagged and pupil diameter in head-fixed mice trained to perform a tactile detection task. Although pupil diameter was found to have a positive and monotonic relationship with LC spiking activity, they found that identical optical LC stimulations evoked variable pupil responses on each trial (Megemont et al., 2022). This variability in the LC-pupil coupling may be linked to the involvement of other brain areas or neuromodulatory systems in controlling pupil fluctuations (Joshi et al., 2016; Reimer et al., 2016; Figures 2C-E). For example, sustained activity in cholinergic axons is observed during longer-lasting pupil dilations, such as those occurring during locomotion (Reimer et al., 2016; Figures 2D, E). In addition, phasic stimulation of the dorsal raphe serotoninergic nuclei can also regulate pupil size and reactivity to sensory stimulation (Cazettes et al., 2021). Other studies suggest that pupil fluctuations can be influenced by hormonal changes (Leknes et al., 2013; Prehn et al., 2013). These factors must be taken into account when interpreting results, in particular in non-drug-free clinical populations. Several medications and drugs of abuse [such as selective serotonin reuptake inhibitors (SSRI) and Opioids] may affect pupillary size and spontaneous fluctuations (Schmitt et al., 2002; Dhingra et al., 2019).

Task-related variables can also influence pupillary variability. Pupil dilation can occur in response to unexpected stimuli (orienting response), expectation violation, and various cognitive processes such as attention, memory load, and decision making (Kahneman and Beatty, 1966; Qiyuan et al., 1985; Alnæs et al., 2014; de Gee et al., 2014; Wang and Munoz, 2014; Figure 2C). Transient pupil dilations are typically linked to phasic LC firing (Aston-Jones et al., 1994), but some factors, such as stimulus salience, are associated with shifts of attention and likely also related to superior colliculus activation (Wang et al., 2012). Additionally, other cortical regions like the anterior cingulate cortex and the orbitofrontal cortex are involved in pupil dynamics (Hayden et al., 2011; Padoa-Schioppa and Conen, 2017). However, LC activation reliably anticipates changes in pupil diameter with an early latency compared with other regions showing a similar relationship with pupil size (Joshi et al., 2016). The interconnectivity between these regions and the LC suggests that fluctuations in pupil size could be a result of the LC regulation of neural activity across certain areas of the brain.

## Pupillometry as a quantitative measure of cortical plasticity

### Noradrenergic role in cortical plasticity

Norepinephrine has been extensively studied in the cortical plasticity framework (Kasamatsu and Pettigrew, 1976; Bear and Daniels, 1983; Marzo et al., 2009; Shepard et al., 2015), starting from the discovery that NE plays a pivotal role in the developmental plasticity of the visual cortex (Kasamatsu and Pettigrew, 1976, 1979; Kasamatsu et al., 1981). Monocular deprivation is an experimental paradigm often used to study visual cortex plasticity in mammals (Nys et al., 2015). In the binocular primary visual cortex (V1), the neuronal response to a stimulus presented to the contralateral eye is significantly greater compared to that of the ipsilateral eye. In the critical period, occluding the contralateral eye leads to a prompt decrease in the level of responsiveness of V1 cells to stimulation of the contralateral eye (Gordon and Stryker, 1996). Kasamatsu and Pettigrew (1976, 1979) first investigated the role of NE in visual cortex plasticity using monocular deprivation (Kasamatsu et al., 1981). They showed that the infusion of 6-hydroxydopamine (6-OHDA), a neurotoxin that destroys noradrenergic terminals in the visual cortex of kittens, abolished ocular dominance plasticity. Monocular deprivation in kittens causes an ocular dominance shift toward the non-deprived eye (Hubel and Wiesel, 1962; Wiesel and Hubel, 1963). However, when the deprived eye is re-opened after a short period of monocular deprivation, neurons in the visual cortex will gradually become binocular again (Hubel and Wiesel, 1962). The intracortical infusion of NE was able to accelerate the recovery of binocular cortical neurons from the effects of a brief monocular deprivation (Kasamatsu et al., 1981). Moreover, in kittens in which the visual cortex has been rendered aplastic by injections of 6-OHDA or propranolol, an antagonist of β-adrenergic receptors (Shirokawa and Kasamatsu, 1987) NE restored ocular dominance plasticity (Pettigrew and Kasamatsu, 1978).

In the visual cortex, parvalbumin inhibitory interneurons (PV) regulate the closure of developmental critical periods and modulate experience-dependent plasticity in adulthood (Fagiolini and Hensch, 2000). It has been demonstrated that the PV cells firing rate is linked with the behavioral state and can be modulated by the release of acetylcholine (ACh) and NE (Garcia-Junco-Clemente et al., 2019). In particular, PV neurons establish functionally distinct subnetworks in the neocortex. During locomotion, the activity of basal forebrain cortical projections can independently modulate the responses of these subnetworks. This modulation occurs through the release of ACh, which suppresses the activity of one group of PV cells, and by NE released from the locus coeruleus during periods of heightened arousal, which enhances the activity of the other group of PV cells. According to the neuromodulatory control of PV activity, it is also possible to distinguish the two functional subnetworks of PV cells by looking at locomotion and pupil diameter (Garcia-Junco-Clemente et al., 2019).

The establishment of long-term plasticity in the cortex necessitates the presence of sensory experience and the involvement of neuromodulatory systems that transmit information about behavioral context to local cortical circuits (Shulz et al., 2000; Froemke et al., 2007; Constantinople and Bruno, 2011). Plasticity in the LC requires the activation of NMDA receptors and can be induced by coupling tones with the depolarization of LC single neurons (Martins and Froemke, 2015). Martins and Froemke (2015) demonstrated that LC plasticity is necessary and sufficient for the induction and maintenance of cortical plasticity in rats primary auditory cortex. In particular, the LC pairing induced an increased response to all the tones across the tuning curve and a long-lasting shift of the tuning curve in the primary auditory cortex (A1). Moreover, they found that LC pairing improved auditory perception in an operant conditioning task. The authors conditioned rats to nose-poke to obtain food rewards in response to 4-kHz target stimuli at different intensities. After pairing the tone at 30 dB with the LC stimulation, the response rate for the 20–40 dB stimuli was enhanced. LC pairing also promoted the ability to distinguish between target and confounding stimuli and accelerate reverse learning when the rewarded tone was changed (Martins and Froemke, 2015). These findings demonstrate that LC plasticity is critical for facilitating the rapid onset and enduring persistence of cortical alterations as a consequence of modifications in brain state and behavior, such as those observed in one-trial learning or post-traumatic stress disorder.

The diffused release of cortical NE to the extracellular space allows NE to act on different cell targets simultaneously, integrating and coordinating multiple cellular and molecular responses. It is becoming evident that microglial cells play an important role as an integral part of the synapse in addition to the neuronal pre-and post-synaptic compartments and astrocytes (Schafer et al., 2013). Microglia are the innate immune cells and phagocytes of the CNS (Tremblay et al., 2010). In the context of injury or disease, microglial cells exhibit a high sensitivity to perturbations in brain homeostasis and are capable of rapid morphological changes in response to inflammatory signals (Dheen et al., 2007). Within the quad-partite synapse, microglial processes continuously survey their environment and establish interactions with other neural cell types such as neurons and astrocytes (Schafer et al., 2013), thereby influencing synaptic remodeling and neural plasticity through the secretion of growth factors, enzymes, and physical contacts with synaptic structures (Schafer et al., 2012; Sipe et al., 2016). Notably, microglia display a distinct and prominent expression of the β2-adrenergic receptor (β2-AR) in the non-injured brain, which distinguishes them from other cell types in the CNS (Zhang et al., 2014). These findings imply that microglial cells may exhibit distinct responses to NE, which potently modulates processes such as plasticity, learning, sensory processing, and attention to salient stimuli (Yang et al., 2014). Stowell et al. (2019) demonstrated that cortical NE release is necessary for microglia morphology changes through β2-ARs. During wakefulness, NE suppresses the branching and movement of microglial cells, and the inhibition of the β2-AR signaling results in an increase of microglial process branching and surveillance, mimicking the effects of anesthesia (Stowell et al., 2019). The activation of β2-AR signaling not only reduces microglial surveillance in the basal state but also attenuates microglial responses to sudden injuries (Stowell et al., 2019). Furthermore, the activation of β2-AR interferes with ocular dominance plasticity and microglial interactions with dendritic spines, showing the critical roles of β2-AR signaling and microglia in modulating experience-dependent plasticity (Stowell et al., 2019).

These studies support the role of the LC-NE system in enhancing cortical plasticity by acting on multiple cell targets. Through increasing the level of waking and arousal, the LC-NE system may participate in information processing, modulating sensory collections and high-order cognition.

### Exploring the relationship between pupil size and visual cortical plasticity

Since pupil dilations coincide with changes in neuromodulatory signaling, pupillometry appears to be a promising technique for estimating the degree of residual plasticity. In a recent study conducted on humans, Binda and Lunghi (2017) demonstrated that monocular deprivation affects spontaneous slow pupil oscillation at rest, called hippus (Diamond, 2001). The authors assessed pupillary oscillations prior to and subsequent to monocular deprivation and observed a heightened amplitude of hippus following visual deprivation. Additionally, individuals with more prominent pupillary fluctuations exhibited more robust alterations in ocular dominance during binocular rivalry dynamics. In a binocular rivalry experiment, incompatible images are presented to each eye simultaneously, but instead of perceiving a combination of the two images, people typically experience slow and irregular perceptual alternations of the two stimuli. The binocular rivalry has become an essential index to study ocular dominance plasticity in humans (Steinwurzel et al., 2020). Because pupil dilations can non-invasively convey NE release, pupil size has been used to study switches between alternative percepts.

Previous findings suggest the occurrence of a transient dilation of the pupil during perceptual switching, which may indicate an increase in NE levels (Einhäuser et al., 2008; de Hollander et al., 2018). In a recent study, Brascamp et al. (2021) reported that changes in perception were accompanied by a complex pupillary response that could be deconstructed into two components: a dilation linked to task execution, plausibly reflecting an arousal-mediated NE increase, and a concurrent constriction associated with the perceptual transition, plausibly indicating alterations in visual cortical representation. The amplitude of constriction, but not dilation, was systematically modulated by the duration between perceptual changes, offering a possible overt measure of neural adaptation (Brascamp et al., 2021). The findings indicate that the size of the pupil reflects the activity of interacting but dissociable neural mechanisms during perceptual multistability and imply that the release of arousal-related neuromodulators affects behavior but not perception.

Different studies support the involvement of NE in modulating homeostatic plasticity, highlighting the potential utility of pupillary fluctuations as a proxy for studying visual cortical plasticity in humans. NE serves as a common source for this phenomena, due to its established role in regulating both pupil diameter modulation (Joshi et al., 2016) and visual cortical plasticity (Kasamatsu and Pettigrew, 1979; Kasamatsu et al., 1981).

In the mouse, the oscillations of pupil constriction and dilation provide an efficient means of monitoring the cortex’s reaction to sensory stimuli (Reimer et al., 2014; Lee and Margolis, 2016). Specifically, the dilation of the pupil is associated with desynchronized activity within neural populations and heightened sensitivity toward visual/somatosensory stimulation, which are both synchronized with the alteration of activity in various categories of inhibitory interneurons (Reimer et al., 2014). These responses are also linked to signaling within the NE and ACh systems (Reimer et al., 2016). Jordan and Keller (2023) recently have demonstrated that the LC-NE system in mice is involved in prediction errors and that LC activity promotes learning by contributing to sensorimotor cortical plasticity. The study also found a significant correlation between LC axon activation in different somatosensory cortical regions and changes in pupil size (Jordan and Keller, 2023).

The close association between NE tone and pupil diameter demonstrates the potential of pupillometry as a valuable tool to study adult cortical plasticity in clinical populations.

## Pupil size as a promising biomarker for brain diseases

The dysregulation of the LC-NE system has been linked to the development of various brain disorders. Decreased noradrenergic activity, for instance, has been observed in individuals with depression (Brunello et al., 2002). Conversely, an increase in noradrenergic activity has been observed in patients with anxiety (Vismara et al., 2020). Furthermore, the LC-NE system also plays a role in the pathogenesis of other brain disorders such as post-traumatic stress disorder, schizophrenia, substance abuse, and neurodegenerative conditions like Alzheimer’s disease (Weinshenker and Schroeder, 2006; Fitzgerald, 2014; Hendrickson et al., 2018; David and Malhotra, 2022). In the following section we will explore the potential role of the LC-NE system in neurodevelopmental disorders characterized by abnormal brain plasticity and its assessment using pupillometry as a non-invasive biomarker.

### Pupil alterations in neurodevelopmental disorders

Brain development and maturation require incredible plasticity. Such plasticity is particularly pronounced during critical periods, specific temporal windows during which the neural circuitry is highly sensitive to both internal and external modulations (Wiesel and Hubel, 1963; Barkat et al., 2011). The importance of NE in regulating neural development (Lovell, 1982; Gustafson and Moore, 1987) is supported by studies that have shown noradrenergic fibers developing prior to the emergence of cortical neurons in the cerebral and cerebellar cortices (Lauder and Bloom, 1974; Sievers et al., 1981; Kolk and Rakic, 2022). During brain development, NE participates in the shaping and wiring of the nervous system (Felten et al., 1982; Gustafson and Moore, 1987; Golovin and Ward, 2016) by creating an opportunity for early life experiences to influence neuronal circuits and cause permanent changes in performance (Herlenius and Lagercrantz, 2004). Early alterations in NE transmission have significant implications for behavior, cognition, and mental health across the lifespan. In rodents, for instance, modifications in the expression of critical genes that regulate NE transmission during vulnerable developmental stages can affect adult circuits involved in emotional behavior, leading to the emergence of anxiety and depression-like symptoms later in life (Schramm et al., 2001; Lähdesmäki et al., 2002; Shishkina et al., 2002, 2004).

An increasing body of studies indicates that pupillometry has the potential to serve as a biomarker for various neurological and psychiatric conditions in both early development and adult populations (Blaser et al., 2014; Frost et al., 2017; Chougule et al., 2019; Iadanza et al., 2020; Winston et al., 2020; El Ahmadieh et al., 2021). For example, the PLR in infancy can predict the severity of autism spectrum disorders (ASDs) (Nyström et al., 2018). In children with ASD, the degree of relative constriction (but not latency) is associated with the extent of sensory dysfunction (Nyström et al., 2018) and infants with a high risk for ASD demonstrated larger PLR compared to low-risk control infants with no family history of ASD. This study shows a significant role of abnormal sensory processing in the etiology of ASD, and proposes that measuring changes in the size of the pupils may aid in identifying infants at risk for ASD. Recent studies also suggest that pupil size may be a potential biomarker for attentional states in individuals with attention-deficit/hyperactivity disorder (ADHD), due to the central role of the LC-NE system in regulating attention (Wainstein et al., 2017). Pupil size variations have been found to indicate alterations in performance during a visuospatial working memory task, which is typically impaired in ADHD patients (Wainstein et al., 2017). Additionally, changes in pupil size have been observed during the presentation of attentionally relevant cues, and have been shown to correlate with individual performance variability and the administration of methylphenidate (Wainstein et al., 2017).

The development of mouse models of neurodevelopmental disorders is a crucial aspect for understanding the molecular and cellular mechanisms involved in brain development, as well as how genetic variance can impact the development of the CNS. Animal models make it possible to study in depth the molecular pathways involved in the pupillary alterations observed in patients. In a recent study, Artoni et al. (2020) reported that mouse models of idiopathic or monogenic ASD display a signature of broadly distributed pupil sizes. Moreover, they have shown that in cholinergic circuits the selective expression of MeCP2 could rescue the pupillary deficit of MeCP2-deficient mice. Despite a direct involvement of neuropathological changes of the LC-NE system in ASD remain controversial, there is numerous evidence that supports the presence of autonomic dysregulation. Recently, we assessed the presence of pupillary abnormalities in a mouse model of cyclin-dependent kinase-like 5 (Cdkl5) deficiency disorder (CDD), a severe neurodevelopmental disorder characterized by early-onset seizures, intellectual disability, motor and cortical visual impairment (Weaving et al., 2004; Moseley et al., 2012). We found in both male and female mutant mice hyperactivity associated with impairment in processes controlling general arousal by measuring pupil size and locomotor behavior (Viglione et al., 2022). We found that Cdkl5 mutants stay longer than wild-type mice in a high arousal state characterized by a dilated pupil and running, they also show alterations in pupillary response during an orienting response visual task (Cohen and Douglas, 1972; Harris et al., 1999; Boxhoorn et al., 2020). These data reveal a global defect in arousal modulation in CDD mice opening to further investigations about the role of NE in neurodevelopmental disorders.

## Conclusion

Pupil dilations have been associated with changes in neuromodulatory signaling, specifically within the NE system, which plays a critical role in regulating pupil diameter modulation and visual cortical plasticity. Recent research suggests that pupillometry is a promising technique for assessing residual plasticity in both humans and mice. Furthermore, early changes in NE transmission can have significant implications for behavior, cognition, and mental health throughout the lifespan. Pupillometry has the potential to serve as a biomarker for various neurological and psychiatric disorders in both early development and adult populations. The evaluation of pupillary abnormalities in mouse models of neurodevelopmental disorders indicates the potential involvement of NE in their pathogenesis and highlights avenues for further investigation. Recent advancements in pupillometry have facilitated the measurement of pupillary responses using commercially available eye trackers and open-source tools. The development of pupillometry tools has also enabled measurements in freely moving animals, allowing for research under more ecologically relevant conditions while performing multiple physiological recordings. The advancement of neuroscience techniques is essential to expand our current knowledge on the LC-NE system in plasticity and pupil size. Additionally, pupillometry has demonstrated potential in telemedicine studies, allowing for studies on how environmental factors affect pupil-based biomarkers.

## Author contributions

AV and RM wrote the initial draft. AV made all figures. All authors discussed the content and commented on the text and figures.
